# Increased Rate of Anemia and Discontinuation in Older Patients with Myelofibrosis Treated with Ruxolitinib

**DOI:** 10.3390/jcm14196811

**Published:** 2025-09-26

**Authors:** Alessandro Laganà, Emilia Scalzulli, Ida Carmosino, Maria Laura Bisegna, Claudia Ielo, Costanza Andriola, Maurizio Martelli, Massimo Breccia

**Affiliations:** Hematology, Department of Translational and Precision Medicine, Policlinico Umberto I, Sapienza University, 00161 Rome, Italy; lagana@bce.uniroma1.it (A.L.);

**Keywords:** Myelofibrosis (MF), young, old, elderly, Ruxolitinib (RUX), age, spleen response (SR)

## Abstract

**Background/Objectives**: Myelofibrosis (MF) predominantly affects older individuals, and its incidence increases with age. Ruxolitinib (RUX), a JAK1/2 inhibitor, effectively reduces spleen volume and relieves disease-related symptoms in MF patients and can be prescribed regardless of age. Although advanced age is associated with poorer MF prognosis, the influence of patient age on RUX treatment efficacy and safety has not been fully elucidated. **Methods**: In this single-center, retrospective study, we included 216 adult MF patients who initiated RUX therapy between 2012 and 2024. Patients were stratified by age at the start of RUX as follows: <65 (*n* = 105), 65–74 (*n* = 64), and ≥75 years (*n* = 47). Clinical data were analyzed in order to assess the impact of age on RUX-associated responses, toxicities, and survival. **Results**: Compared to younger patients, those ≥65 years showed features of more advanced MF and 45% higher odds of not achieving SR [OR = 1.45 (95% CI, 1.10–1.91), *p* = 0.009]. Patients ≥65 years presented a higher incidence of drug-related anemia at 3 (*p* = 0.003) and 6 months (*p* = 0.020). These patients had a two-fold increased risk of RUX discontinuation [HR = 2.07 (95% CI, 1.30–3.31) (*p* = 0.002) and presented a shorter OS than younger patients [HR = 2.74 (95% CI, 1.67–4.49)] (*p* < 0.001). In the sub-analysis focused on patients older than 65 years, very elderly patients (≥75 years) exhibited similar baseline characteristics, SR rates, median RUX treatment duration (*p* = 0.22), and OS (*p* = 0.86) to the 65–74 years cohort. More patients in the very elderly group presented an infectious event grade ≥ 2 (19.2%) than in the 65–74 years group (3.1%) (*p* = 0.008). **Conclusions**: RUX demonstrates overall robust rates of SR and favorable OS across all age groups. However, patients aged ≥65 years experienced higher rates of adverse events and worse outcomes. Our data support RUX usage in all age cohorts while highlighting the need for tailored strategies and close clinical monitoring in older patients.

## 1. Introduction

Myelofibrosis (MF), including both primary MF (PMF) and secondary MF (SMF) to essential thrombocythemia (ET) or polycythemia vera (PV), is a Philadelphia-negative myeloproliferative neoplasm (MPN) associated with progressive bone marrow (BM) fibrosis, splenomegaly, and constitutional symptoms [1]. The dysregulation of the JAK/STAT pathway, primarily driven by mutations in key “driver” genes (JAK2, CALR, and MPL), is the pivotal molecular alteration characterizing MPNs and MF [2]. MF is a rare disease, with a worldwide annual incidence ranging from 0.22 to 0.99 cases per 100,000 persons [3]. Although MF can occur at any age, it is predominantly considered a disease affecting older adults, typically diagnosed in individuals over 50 with a median age at diagnosis of approximately 65 years. The incidence increases markedly with advancing age. While around 30% of cases occur in those aged 65 to 74, over half of patients are diagnosed after the age of 75 [1,4].

Older age is widely recognized as a key risk factor associated with poorer outcomes in MPN patients, including reduced overall survival (OS) and an increased risk of progression to acute myeloid leukemia (AML) [5]. In MF, although different prognostic score risk models introduce different clinical and laboratory parameters, most emphasize advanced age as a critical determinant of poor prognosis. For instance, the International Prognostic Scoring System (IPSS) [6] and the Dynamic International Prognostic Scoring System (DIPSS) [7] both use 65 years as a cutoff to identify patients with worse prognosis. In some cases, advanced age alone can shift patients into higher risk categories, underscoring its importance as an independent prognostic factor for MF management.

In the past 10–15 years, the approval of small-molecule Janus kinase inhibitors (JAKi) has significantly reshaped therapeutic strategies for MF [8]. Ruxolitinib (RUX), a JAK1/JAK2 inhibitor, represents the first approved and most widely used JAKi in clinical practice. It has demonstrated significant clinical benefits by reducing spleen volume and alleviating the debilitating MF symptoms in patients with intermediate- or high-risk disease features [9,10,11,12]. Although early data regarding the impact of RUX on OS were discordant in some cases [13], more recent evidence suggests that RUX may indeed provide a survival benefit [14,15]. Pivotal RUX trials included both older and very elderly patients, with a relatively high median age [9,10]. Nevertheless, few specific subgroup analyses were conducted to assess the efficacy and safety of RUX, specifically in older and very elderly populations. In fact, despite its proven clinical benefits, RUX has been associated with dose-dependent adverse events (AEs), which may occur more frequently and be more severe in older patients [16].

Hence, the aim of our retrospective study was to evaluate disease features, baseline clinical characteristics, and RUX treatment patterns according to age at RUX initiation. In addition, we sought to assess AE rates and clinical outcomes in these RUX-treated MF patients, stratified by age, in a real-world clinical practice setting.

## 2. Materials and Methods

### 2.1. Patients and Study Design

This retrospective single-center study analyzed records of MF patients who were treated with RUX (Jakavi^®^, Novartis Pharma AG, Basel, Switzerland) between 2012 and 2024. Eligibility for RUX was determined based on the current Italian indications and reimbursement policies for RUX administration in MF: age ≥ 18 years; a diagnosis of either PMF (including early MF with splenomegaly > 5 cm below left costal margin), post-PV SMF, or post-ET SMF according to World Health Organization (WHO) criteria [17]; an IPSS [6] and/or DIPSS [7] intermediate-1 risk or higher; palpable spleen measuring ≥ 5 cm below the left costal margin (LCM); and platelet (PLT) count ≥ 50 × 10^9^/L. Patients who had started RUX less than 6 months before the date of data cutoff were not included in the study. However, patients who experienced early death or other early events within the first 6 months of treatment were included in the analysis (Supplementary Figure S1). Decisions regarding RUX initiation, dose adjustments, and discontinuation were made according to institutional approach. Each patient was followed until death or to data cutoff (31 December 2024). Informed consent for both treatment and the use of clinical data for research was obtained from all patients. This study was conducted in accordance with the ethical standards of the Declaration of Helsinki.

### 2.2. Patients Assessment and Definitions of Terminology

At baseline, patients were stratified into two primary age groups based on their age at the start of RUX: those aged <65 years and those aged ≥65 years (older group). Among patients aged ≥65 years, further subgroup analyses were performed, comparing those aged 65–74 years with those aged ≥75 years (very elderly group). This stratification aimed to evaluate age-related differences in response and tolerability to RUX.

Baseline anemia and thrombocytopenia were defined as a hemoglobin (Hb) level of <10 g/dL and a platelet count of <100 × 10^9^/L before RUX initiation, respectively [18,19,20,21]. Prior to the start of treatment, other possible causes of anemia (such as iron, folate or cobalamin deficiencies, and gastrointestinal bleeding) and of thrombocytopenia (such as autoimmune diseases and viral infections) were ruled out. Baseline or subsequent constitutional symptoms were recorded as absent or present. The term “red blood cell (RBC) transfusion dependency” was defined as the requirement for any RBC transfusions during the three months prior to the specified time point.

Following baseline assessment, patients underwent regular outpatient evaluations approximately every 2–3 months, with additional visits scheduled as needed based on clinical condition. Each evaluation included a physical examination and routine laboratory tests, with imaging studies carried out when clinically appropriate. Spleen size was measured by physical examination, with splenic length (in centimeters—cm) below the LCM recorded at every visit in a real-life clinical setting.

### 2.3. Outcome Measures

Drug-related anemia was defined as new or worsening anemia characterized either by a drop in Hb levels below 10 g/dL, an increase of at least one grade from baseline, or the new onset of a requirement for RBC transfusions [19,20,21]. Emergent (or drug-related) thrombocytopenia was defined as new or worsening thrombocytopenia with PLTs < 100 × 10^9^/L and a higher grade than baseline [22,23]. Grading of anemia and thrombocytopenia was based on the National Cancer Institute Common Terminology Criteria for Adverse Events (NCI-CTCAE) v5.0. All infections grade ≥ 2 according to NCI-CTCAE were recorded (including bacterial, viral, and fungal episodes).

Splenic response was evaluated based on the criteria outlined in the 2013 International Working Group on Myelofibrosis Research and Treatment (IWG-MRT)/European LeukemiaNet (ELN) consensus report [24]. Spleen response duration was defined as the time from the first documentation of splenic response to either loss of criteria for spleen response or last follow-up. Response to Ruxolitinib after 6 months (RR6) was calculated with clinical data obtained during the first 6 months of follow-up [25]. Diagnosis of transformation to AML was made according to the WHO criteria [17]. Overall survival (OS) was defined as the time from the initiation of RUX treatment to death from any cause or to the date of the last follow-up.

### 2.4. Statistical Analysis

Normality of continuous variables was assessed using the Shapiro–Wilk test. Due to non-normal distribution of most continuous variables, they are reported as medians with interquartile ranges (IQR) and/or overall ranges. Categorical variables are presented as frequencies (*n*) and expressed as percentages and/or ratios. Differences between study groups were estimated using the Pearson chi-square test or the Fisher exact test for categorical covariates and the Mann–Whitney U test or Kruskal–Wallis test for continuous variables. The association between two continuous variables was examined using Pearson’s correlation coefficient or Spearman’s rank correlation coefficient, as appropriate. Probability of OS was estimated using Kaplan–Meier analysis. RUX discontinuation analysis was performed by means of Kaplan–Meier curves. Log-rank Mantel–Cox on Kaplan–Meyer curves was used to assess statistical significance. Cox proportional hazards regression model was used to determine the effect of age on OS and RUX discontinuation. The odds ratio (OR) and hazard ratio (HR) for each independent variable were determined with a confidence interval (CI) of 95%. All tests were two-sided, with *p* values < 0.05 considered statistically significant. All statistical analyses were performed using IBM SPSS Statistics, version 26.0 (IBM Corp., Armonk, NY, USA).

## 3. Results

### 3.1. Population and General Characteristics

In total, 216 RUX-treated MF patients were included in the analysis, and their characteristics are shown in Table 1. The overall median age at the start of RUX treatment was 66.1 years (IQR, 56.1–73.7), with 111 male (51.4%) and 105 female (48.6%) patients. PMF accounted for 98 (45.4%) cases, while of the 118 patients with SMF (54.6%), 62 (28.7%) had ET-SMF and 56 (25.9%) had PV-SMF, respectively. Based on the driver mutation profile, 163 (75.5%) patients harbored a JAK2 V617F mutation, 20 (9.3%) had CALR mutations (type 1/1-like or type 2/2-like), two (0.9%) had MPL mutations, and 13 (6.0%) had triple-negative MF, while the other 18 cases, who were JAK2 (V617F)-negative, were not tested for CALR and/or MPL mutations. At the start of RUX, 35 (16.2%) patients presented early MF and 181 (83.8%) presented overt MF. The median time from MF diagnosis to RUX initiation was 5.6 months (IQR, 1.5–43.5). All patients were JAKi naïve, while 115 (53.2%) had received at least one other previous line of treatment. Based on IPSS, patients were stratified as follows: 72 (33.3%) as intermediate-1, 63 (29.2%) as intermediate-2, and 81 (37.5%) as high risk. DIPSS risk stratification showed that 111 (51.4%) were classified as intermediate-1, 75 (34.7%) as intermediate-2, and 30 (13.9%) as high risk. Overall, at baseline, the median Hb value was 11.1 g/dL (IQR, 9.5–13.0), the median PLT count was 279.5 × 10^9^/L (IQR, 158.0–489.0), and the median white blood cell (WBC) count was 10.5 × 10^9^/L (IQR, 6.3–16.7). There was a significant decrease in Hb (r = −0.33, *p* < 0.001) and PLTs (r = −0.19, *p* = 0.005) in older patients, while there was a significant increase in blast-cells in PB (ρ = 0.21, *p* = 0.002). Lactate dehydrogenase (LDH) levels were ≥2 times the upper normal limit (ULN) in 144 patients (66.7%), and 173 (80.1%) presented at least one constitutional symptom at baseline. The initial RUX dosage was distributed as follows: 20 mg of BID in 119 patients (55.1%), 15 mg of BID in 39 (18.1%), 10 mg of BID in 34 (15.7%), and 5 mg of BID in 24 patients (11.1%).

### 3.2. Stratification by Age: Under 65 vs. Over 65

#### 3.2.1. Baseline Characteristics

A total of 111 patients (51.4%) were aged 65 years or older at the start of RUX. Compared to younger patients, those ≥ 65 years showed features of a more advanced MF (Table 1). Higher grades of BM fibrosis (*p* = 0.032) and, consequently, a greater prevalence of overt MF (89.2% vs. 78.1%—*p* = 0.041) were evident in older patients. At baseline, the median Hb [10.3 g/dl (IQR, 8.9–12.0) vs. 12.0 g/dl (IQR, 9.9–14.0)—*p* < 0.001] and PLT counts [250.0 × 10^9^/L (IQR, 154.0–438.0) vs. 333.0 × 10^9^/L (IQR, 175.5–535.0)—*p* = 0.026] were lower in patients ≥ 65 years; thus, baseline anemia was more common (46.8% vs. 26.7%, *p* = 0.003), along with significant RBC dependence at the time of RUX initiation (24.3% vs. 11.4%, *p* = 0.021) in older patients. LDH levels ≥ 2 ULN were more frequent (74.8% vs. 58.1%—*p* = 0.014), and the median percentage of blast-cells in PB was higher [1.0% (IQR, 0.0–2.0) vs. 0.0 (IQR, 0.0–1.0)—*p* = 0.027] in the older group. Patients ≥ 65 years were predominantly classified in the higher IPSS and DIPSS risk classes compared to those < 65 years (*p* < 0.001). Even when classified by the age-adjusted DIPSS (aaDIPSS) score, avoiding the age-related penalty in standard DIPSS, patients of the older group were significantly more likely to fall into higher risk classes (*p* < 0.001). However, notably, a higher percentage of patients < 65 years presented with at least one constitutional symptom before the start of RUX (89.5% vs. 71.2%, *p* = 0.001). A significant difference in initial RUX dosing was observed between the age groups. In the younger cohort, 69.6% of patients started at 20 mg BID, whereas the older cohort displayed a more heterogeneous dosing pattern: 41.4% at 20 mg BID, 24.3% at 15 mg BID, 19.8% at 10 mg BID, and 14.5% at 5 mg BID (*p* = 0.003). Moreover, patients ≥65 years were more frequently started on a lower-than-expected initial RUX dose based on their PLT count compared to younger patients (35.1% vs. 14.3%, *p* < 0.001).

#### 3.2.2. Spleen and Symptoms Response, RUX Dose Reduction, and RR6

During the entire follow-up, 99 (45.8%) patients achieved a spleen response (SR) after a median RUX treatment period of 0.9 months (IQR, 0.5–1.6). Patients aged ≥ 65 years showed a lower overall SR rate compared to younger patients (36.9% vs. 55.2%), with approximately 1.5-fold higher odds of not achieving SR [OR = 1.45 (95% CI, 1.10–1.91), *p* = 0.009]. SR duration was also significantly shorter in the older group (25.4 vs. 34.5 months, *p* = 0.045). Analogously, at 3 and 6 months, SR was achieved by 32.4% and 34.2% of patients ≥ 65 years, respectively, compared to 46.2% and 50.5% in the younger patients. Older patients had higher odds of not achieving SR, with an OR of 1.34 (95% CI, 1.01–1.78) (*p* = 0.049) at 3 months and 1.39 (95% CI, 1.05–1.85) (*p* = 0.019) at 6 months.

Similarly, among patients with baseline MF-related symptoms, improvement at 6 months was more frequent in younger individuals (80.4%) compared with those aged ≥65 years (57.0%) (*p* = 0.001).

Although there was an overall reduction in RUX dose during the first 6 months of treatment, the percentage decrease observed at 3 and 6 months was similar in both age groups. However, as at baseline, at 3 months (*p* = 0.024) and 6 months (*p* = 0.007), patients aged ≥65 years received significantly lower RUX doses (Supplementary Table S1). Consequently, stratified at 6 months by RR6 score, patients in the older group were significantly more often classified into higher risk categories than young patients: 19.9% (n = 22) were low risk vs. 52.4% (n = 55); 47.7% (n = 53) vs. 29.5% (n = 31) were intermediate risk; and 32.4% (n = 36) vs. 18.1% (n = 19) were high risk (*p* < 0.001).

#### 3.2.3. RUX-Related Toxicity

Overall, 78 (36.1%) and 71 (32.9%) patients developed drug-related anemia at 3 and 6 months during RUX therapy, respectively. Among patients ≥ 65 years, the incidence of drug-related anemia was significantly higher than in those < 65 years at both 3 months (45.9% vs. 25.7%) (*p* = 0.003) and 6 months (40.5% vs. 24.7%) (*p* = 0.020). Moreover, of the 177 patients who were not transfusion-dependent at baseline, a higher proportion of patients in the older group developed new RBC transfusion dependency at 3 months [31.0% (26/84) vs. 8.6% (8/93); *p* < 0.001] and at 6 months [28.6% (24/84) vs. 14.0% (13/93); *p* = 0.026] (Table 2).

Overall, 25 (11.6%) and 30 (13.9%) patients had developed drug-related thrombocytopenia at 3 and 6 months during RUX treatment, respectively. The incidence of drug-related thrombocytopenia was comparable in both groups at 3 and 6 months (Table 2). Sixteen infective episodes grade ≥ 2 (7.4%) occurred, with 11 cases developing in patients ≥ 65 years (9.9%) and 5 in patients < 65 years (4.8%) (*p* = 0.20).

#### 3.2.4. RUX Discontinuation and Overall Survival

**Figure 1:**
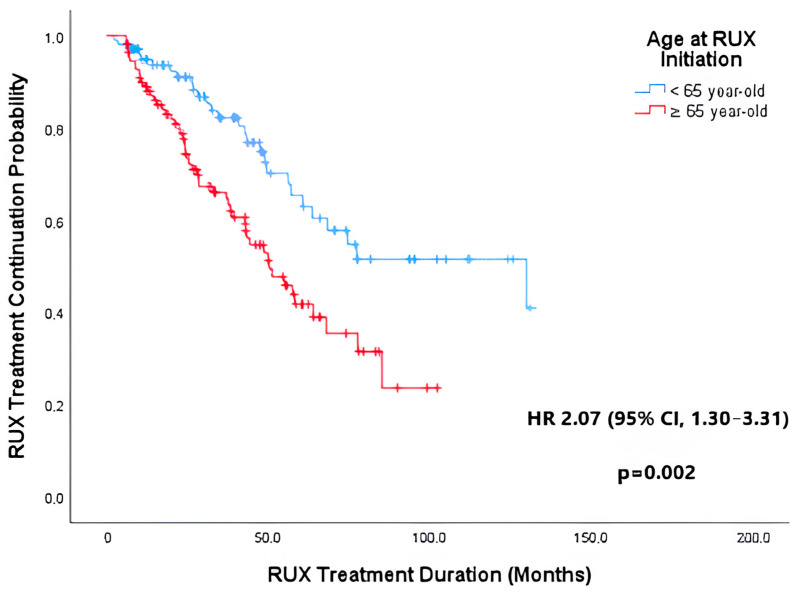
Kaplan–Meier curve for RUX discontinuation in the older group (≥ 65 years) and in the younger group (< 65 years). RUX, ruxolitinib; HR, hazard ratio; CI, confidence interval.

For the outcome analysis, 21 patients (9.7%) who underwent allo-HSCT were censored at the date of transplant, as RUX was discontinued and the procedure might have influenced survival. In our cohort, the median treatment duration was 56.2 months (IQR, 25.5–129.9), and 82 patients (40.0%) discontinued treatment during follow-up for reasons other than allo-HSCT. Major discontinuation reasons were progression to AML in 33 (40.2%) and switch to other JAKi in 24 (29.3%) cases. Patients aged ≥ 65 years were on RUX treatment for a shorter period [median 50.3 months (95% CI, 37.4–63.2) vs. median 129.9 months (95% CI, 53.3–206.4)] and had a two-fold increased risk of RUX discontinuation than younger patients [HR = 2.07 (95% CI, 1.30–3.31)] (*p* = 0.002) (Figure 1).

**Figure 2:**
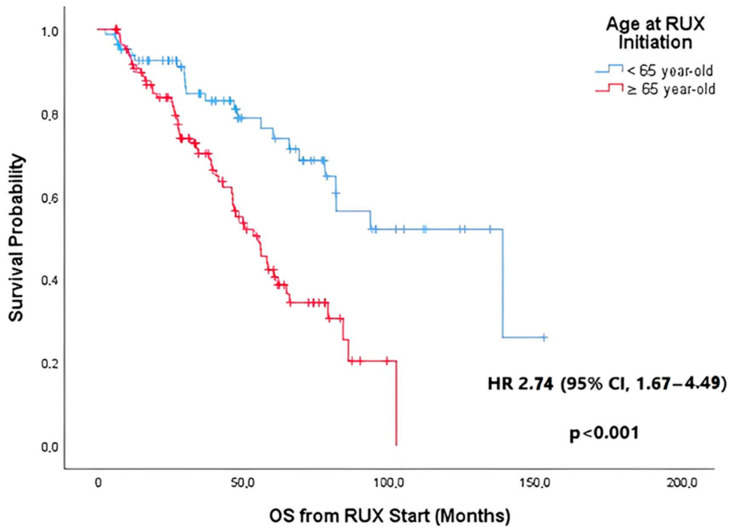
Kaplan–Meier curve for overall survival (OS) probability from ruxolitinib (RUX) initiation in the older group (≥65 years) and in the younger group (<65 years). RUX, ruxolitinib; OS, overall survival; HR, hazard ratio; CI, confidence interval.

After a median follow-up of 61.8 months (IQR, 33.7–94.0), 80 patients (37.0%) died. The leading causes of death were transformation to AML in 37 patients (46.3%) and cardiovascular disease in 13 patients (16.3%). Overall median OS was 69.2 months (95% CI, 52.2–86.2). Patients aged ≥ 65 years presented a shorter OS than younger patients, as shown in Figure 2 [55.3 months (95% CI, 44.7–65.9) vs. 138.7 months (95% CI, 69.3–208.1)] [HR = 2.74 (95% CI, 1.67–4.49)] (*p* < 0.001).

### 3.3. Subgroup Analysis of the Old Cohort: 65–74 vs. ≥75 Years (Very Elderly)

To further elucidate the role of age in elderly patients, a sub-analysis was performed on patients older than 65 years, stratifying them into two cohorts: 65–74 years and ≥75 years (very elderly group) at the moment of RUX initiation.

Among the 111 patients aged 65 years or older, 47 (42.3%) were ≥75 years old. As shown in Table 3, no statistically significant differences in baseline characteristics or MF features were evidenced between the 65–74 and ≥75 years cohorts, and their initial RUX dosages were comparable.

Outcomes in these two groups were comparable as well. During the entire follow-up, SR was obtained by 16 patients (34.0%) in the very elderly group and 25 patients (39.1%) in the cohort aged 65–74 years (*p* = 0.69), with similar median SR durations of 23.0 (IQR, 13.3–60.5) and 27.1 months (IQR, 17.0–42.9), respectively (*p* = 0.65). Likewise, the SR rates at both 3 and 6 months were similar. Moreover, no difference in MF-related symptom alleviation rate at 6 months was evidenced (*p* = 0.25).

Dose reduction rates at 3 and 6 months were comparable between age groups; however, a significantly greater proportion of very elderly patients reduced their RUX dose between the third and sixth months compared to those aged 65–74 (34.0% vs. 10.9%; *p* = 0.004). Nevertheless, RUX doses remained comparable at both time points (Supplementary Table S2), and RR6 stratification showed no difference (*p* = 0.27).

**Figure 3:**
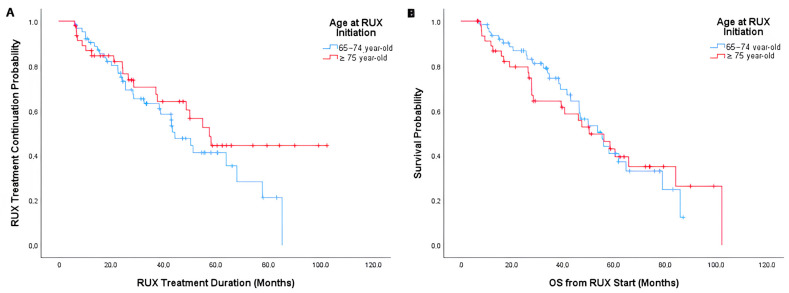
(**A**) Kaplan–Meier curve for RUX discontinuation in the subgroup aged 65–74 years and in the very elderly group (≥75 years). (**B**) Kaplan–Meier curve for OS from RUX start in the subgroup aged 65–74 years and in the very elderly group (≥75 years). RUX, ruxolitinib; OS, overall survival; HR, hazard ratio; CI, confidence interval.

Very elderly patients had a median RUX treatment duration of 57.5 months (95% CI, 45.6–69.4), compared to 44.3 months (95% CI, 34.9–53.8) in the 65–74 cohort, with no significant difference (Figure 3A; *p* = 0.22). Likewise, after a median follow-up of 60.8 months (IQR, 33.3–78.0), the median OS was 50.3 months (95% CI, 34.7–66.0) in the very elderly group and 55.3 months (95% CI, 43.7–66.9) in the 65–74 cohort, with no significant difference (Figure 3B; *p* = 0.86).

The incidence rates of drug-related anemia and thrombocytopenia at 3 and 6 months were similar, but more patients in the very elderly group acquired new RBC transfusion dependency at 6 months than those aged 65–74 years [41.2% (14/34) vs. 20.0% (10/50); *p* = 0.049] (Supplementary Table S3). Moreover, the reported cases of an infectious event grade ≥ 2 were higher in patients ≥ 75 years (9 cases—19.2%) than in patients aged 65–74 years (2 cases—3.1%) (*p* = 0.008).

## 4. Discussion

Since its approval for clinical use, RUX has been broadly adopted for the treatment of MF, with disease incidence increasing with age, thus making it essential to assess RUX-associated outcomes and AEs across different age cohorts. In this study, efficacy and safety data regarding RUX treatment across three age-defined cohorts treated at our center were discussed.

The results showed that RUX determined overall favorable therapeutic outcomes across all age cohorts, with SR rates comparable to those reported in pivotal registration trials [9,10,11,12] and confirmed by other real-world studies [26].

Nevertheless, younger patients (<65-year-old) presented overall better therapeutic outcomes than older patients (≥65-year-old), with higher rates of SR and better OS. These findings may reflect a more advanced disease phenotype at the time of RUX initiation in the older patients. Notably, the older cohort received lower initial RUX doses overall and were more frequently started on a lower-than-expected RUX dose, and this might have influenced prognosis as well. Moreover, patients ≥65 years had a greater incidence of drug-related anemia at 3 and 6 months, with a higher proportion of patients developing new RBC transfusion dependency at the two time points. These findings indicate that older patients are more susceptible to RUX-related AEs, particularly hematological ones. Indeed, patients aged ≥65 years received significantly lower RUX doses at 3 and 6 months and discontinued RUX treatment more frequently during follow-up than younger patients.

Our findings align with the interim results of the Italian ROMEI observational study, which reported that patients over 65 were overrepresented in the lower-than-expected RUX dosage group. This cohort exhibited a poorer estimated median OS compared to those receiving the recommended initial dose, and the authors highlight that an older age should be taken into account because it influences OS [27].

In our sub-analysis focused on patients older than 65 years, the very elderly patients (≥75 years) exhibited baseline characteristics, SR rates, median RUX duration, and OS comparable to the 65–74-year cohort. A higher proportion of patients aged ≥75 years developed new RBC transfusion dependence at six months and experienced more infective events than those aged 65–74. These observations suggest that very elderly patients might be particularly vulnerable to long-term RUX-related AEs, as reflected by a greater frequency of dose reductions between months 3 and 6 compared with the 65–74 cohort.

In 2018, a study by Palandri et al. focused on 291 RUX-treated MF patients that were aged 65 or older at the time of RUX initiation [28]. They reported that the two age cohorts (patients aged 65–74 years and ≥75 years) had comparable baseline characteristics, with the only difference being the significantly lower baseline PLTs in the very elderly patients, resulting in lower RUX starting doses. In terms of toxicity, a higher proportion of very elderly patients presented with anemia (any grade) and/or thrombocytopenia (any grade) during RUX therapy (*p* = 0.04), but the incidence of anemia at 3 and 6 months was not influenced by age, and no differences in RUX dose reductions during the first 3 months were evidenced. They reported a similar cumulative incidence of RUX discontinuation in the two groups. Focusing on efficacy, the overall SR rates and SR at 3 and 6 months were comparable in the two age groups. Nevertheless, the very elderly patients showed a significantly worse OS compared to the patients aged 65–74 years (*p* < 0.001) [28].

Overall, their findings mostly align with our sub-analysis of patients aged ≥65 years, with no significant differences in baseline characteristics and SR and RUX discontinuation rates between very elderly and patients aged 65–74 years. Moreover, the reported toxicity might suggest that very elderly patients have an increased susceptibility to long-term RUX-related AEs. Conversely, Palandri et al. observed a worse OS in their very elderly cohort, an outcome that differs from our analysis, but the median follow-up reported was just 19.5 months, whereas our cohort benefits from 60.8 months of observation in the ≥65-year-old cohort. In the following years, clinicians have accumulated real-world experience that has improved dose optimization and toxicity management, and landmark trials such as JUMP [16,29] and REALISE [30], specifically designed to evaluate RUX safety and efficacy in patients with baseline cytopenias, have further refined practice clinical guidelines. We might hypothesize that collectively, our longer follow-up, enhanced therapeutic expertise, and insights from recent studies may likely account for this discrepancy. In fact, more recent studies show that very elderly patients may have a clear advantage with RUX treatment, and their prognosis has improved drastically [31,32].

Our study is subject to some limitations. Its retrospective, single-center design may not have captured all relevant variables, and sample was limited to patients treated at our center, which may restrict the generalizability of our findings. For instance, despite our rigorous efforts to attribute cytopenias and infections to RUX rather than disease progression, the retrospective nature of the study precludes completely ruling out the contribution of underlying disease evolution to these events. Next-generation sequencing (NGS) data were only available for a subset of patients, preventing analysis of the interplay between age and non-driver mutations, which may have led to worse prognosis. Additionally, although we stratified participants into age cohorts, which could introduce classification bias, treatment choices regarding RUX initiation, dose modifications, and discontinuation were evaluated on a case-by-case basis by treating physicians according to clinical needs.

## 5. Conclusions

Our real-world data confirm that RUX provides meaningful SR and favorable OS across all age groups of MF patients, underscoring that chronological age alone should not preclude RUX use, and no upper age limit should be applied for its administration. Nevertheless, individuals aged ≥65 years are expected to experience a higher incidence of hematological toxicity, an increased likelihood of treatment discontinuation, and a shorter OS compared with younger counterparts. Importantly, focusing on the ≥65-year-old cohorts, RUX discontinuation rates among those ≥75 years were similar to those aged 65–74 years, indicating that with vigilant dose optimization and proactive supportive care, very elderly patients can maintain treatment durability and achieve survival outcomes comparable to cases aged 65–74. These findings underscore the critical influence of aging on both safety and efficacy and support the implementation of age-tailored monitoring and individualized dosing strategies to maximize the benefits of RUX across the full spectrum of patient age.

## Figures and Tables

**Table 1:** Overall cohort characteristics at start of ruxolitinib (RUX) treatment and differences between patients aged ≥ 65 years (older group) and patients < 65 years (younger group). *p* values in bold are statistically significant.

Baseline Characteristics	Total (N = 216)	Age at Start of RUX < 65 Years (*n* = 105)	Age at Start of RUX ≥ 65 Years (*n* = 111)	*p* Value
Median Age (IQR) (years)	66.1 (56.1–73.7)	55.8 (51.0–61.1)	73.6 (70.5–77.2)	**<0.001**
Male (%)	111 (51.4)	52 (49.5)	59 (53.2)	0.68
Female (%)	105 (48.6)	53 (50.5)	52 (46.8)	
PMF (%)	98 (45.4)	41 (39.0)	57 (51.4)	0.19
PV-SMF (%)	56 (25.9)	30 (28.6)	26 (23.4)	
ET-SMF (%)	62 (28.7)	34 (32.4)	28 (25.2)	
Early MF (%)	35 (16.2)	23 (21.9)	12 (10.8)	**0.041**
Overt MF (%)	181 (83.8)	82 (78.1)	99 (89.2)	
Fibrosis Grade 1	35 (16.2)	23 (21.9)	12 (10.8)	**0.032**
Fibrosis Grade 2	65 (30.1)	34 (32.4)	31 (27.9)	
Fibrosis Grade 3	116 (53.7)	48 (45.7)	68 (61.3)	
Untreated (%)	101 (46.8)	57 (54.3)	44 (39.6)	**0.041**
Pre-Treated (%)	115 (53.2)	48 (45.7)	67 (60.4)	
Mutated JAK2 (V617F) (%)	163 (75.5)	78 (74.3)	85 (76.6)	0.75
Wild-Type JAK2 (%)	53 (24.5)	27 (25.7)	26 (23.4)	
Median Hb (g/dl) (IQR)	11.1 (9.5–13.0)	12.0 (9.9–14.0)	10.3 (8.9–12.0)	**<0.001**
Median WBC (×10^9^/L) (IQR)	10.5 (6.3–16.7)	10.0 (6.7–17.4)	10.5 (5.7–15.8)	0.24
Median PMN (×10^9^/L) (IQR)	7.5 (4.0–12.8)	7.8 (4.7–13.7)	6.9 (3.7–11.4)	0.27
Median PLTs (×10^9^/L) (IQR)	279.5 (158.0–489.0)	333.0 (175.5–535.0)	250.0 (154.0–438.0)	**0.026**
Median Blast-Cells in PB (%) (IQR)	0.0 (0.0–1.8)	0.0 (0.0–1.0)	1.0 (0.0–2.0)	**0.027**
Median Spleen Length from LCM (cm) (IQR)	7.0 (6.0–10.0)	8.0 (6.0–10.0)	7.0 (6.0–10.0)	0.15
LDH < 2 ULN	72 (33.3)	44 (41.9)	28 (25.2)	**0.014**
LDH ≥ 2 ULN	144 (66.7)	61 (58.1)	83 (74.8)	
IPSS Intermediate-I (%)	72 (33.3)	60 (57.1)	12 (10.8)	**<0.001**
IPSS Intermediate-II (%)	63 (29.2)	28 (26.7)	35 (31.5)	
IPSS High Risk (%)	81 (37.5)	17 (16.2)	64 (57.7)	
DIPSS Intermediate-I (%)	111 (51.4)	75 (71.4)	36 (32.4)	**<0.001**
DIPSS Intermediate-II (%)	75 (34.7)	26 (24.8)	49 (44.1)	
DIPSS High Risk (%)	30 (13.9)	4 (3.8)	26 (23.4)	
aaDIPSS Low Risk (%)	12 (5.6)	0 (0.0)	12 (10.8)	**<0.001**
aaDIPSS Intermediate-I Risk (%)	96 (44.4)	61 (58.2)	35 (31.5)	
aaDIPSS Intermediate-II Risk (%)	58 (26.9)	27 (25.7)	31 (27.9)	
aaDIPSS High Risk (%)	50 (23.1)	17 (16.2)	33 (29.8)	
Median Time (months) from MF Diagnosis to Start of RUX (IQR)	5.6 (1.5–43.5)	3.3 (1.2–38.6)	7.6 (2.0–48.8)	0.074
RUX Initial Dosage:				**0.003**
5 mg BID (%)	24 (11.1)	8 (7.6)	16 (14.5)	
10 mg BID (%)	34 (15.7)	12 (11.4)	22 (19.8)	
15 mg BID (%)	39 (18.1)	12 (11.4)	27 (24.3)	
20 mg BID (%)	119 (55.1)	73 (69.6)	46 (41.4)	

Abbreviations: RUX, ruxolitinib; PMF, primary myelofibrosis; SMF, secondary myelofibrosis; PV-SMF, post-polycythemia vera MF; ET-SMF, post-essential thrombocythemia MF; Hb, hemoglobin; WBC, white blood cell; PMN, polymorphonuclear neutrophil; PLTs, platelets; PB, peripheral blood; LCM, left costal margin; LDH, lactate dehydrogenase; ULN, upper normal limit; IPSS, International Prognostic Score System; DIPSS, Dynamic International Prognostic Score System; aaDIPSS, age-adjusted Dynamic International Prognostic Score System; BID, twice daily; IQR, interquartile range.

**Table 2:** Distribution of hematological toxicity (drug-related anemia and drug-related thrombocytopenia) at 3 and 6 months from the start of ruxolitinib (RUX) therapy in patients aged under and over 65 years. *p* values in bold are statistically significant.

	Drug-Related Anemia			Drug-Related Thrombocytopenia		
	Age at Start of RUX < 65 Years (*n* = 105)	Age at Start of RUX ≥ 65 Years (*n* = 111)	*p* Value	Age at Start of RUX < 65 Years (*n* = 105)	Age at Start of RUX ≥ 65 Years (*n* = 111)	*p* Value
**At 3 months (%)**	27 (25.7)	51 (45.9)	**0.003**	10 (9.5)	15 (13.5)	0.40
New RBC transfusion dependency at 3 months (%)	8/93 (8.6)	26/84 (31.0)	**<0.001**	-	-	-
**At 6 months (%)**	26 (24.7)	45 (40.5)	**0.020**	13 (12.4)	17 (15.3)	0.69
New RBC transfusion dependency at 6 months (%)	13/93 (14.0)	24/84 (28.6)	**0.026**	-	-	-

**Table 3:** Characteristics at start of ruxolitinib (RUX) treatment in patients aged ≥ 65 years and differences between patients aged 65–74 years and patients ≥ 75 years (very elderly group). *p* values in bold are statistically significant.

Baseline Characteristics	Age at Start of RUX ≥ 65 Years (*n* = 111)	Age at Start of RUX 65–74 Years (*n* = 64)	Age at Start of RUX ≥ 75 Years (*n* = 47)	*p* Value
Median Age (IQR) (years)	73.6 (70.5–77.2)	70.9 (68.6–72.8)	77.7 (75.9–79.9)	**<0.001**
Male (%)	59 (53.2)	32 (50.0)	27 (57.4)	0.45
Female (%)	52 (46.8)	32 (50.0)	20 (42.6)	
PMF (%)	57 (51.4)	31 (48.4)	26 (55.3)	0.45
PV-SMF (%)	26 (23.4)	14 (21.9)	12 (25.5)	
ET-SMF (%)	28 (25.2)	19 (29.7)	9 (19.2)	
Early-MF (%)	12 (10.8)	9 (14.1)	3 (6.4)	0.23
Overt-MF (%)	99 (89.2)	55 (85.9)	44 (93.6)	
Fibrosis Grade 1	12 (10.8)	9 (14.1)	3 (6.4)	038
Fibrosis Grade 2	31 (27.9)	16 (25.0)	15 (31.9)	
Fibrosis Grade 3	68 (61.3)	39 (60.9)	29 (61.7)	
Untreated (%)	44 (39.6)	27 (42.2)	17 (36.2)	0.56
Pre-Treated (%)	67 (60.4)	37 (57.8)	30 (63.8)	
Mutated JAK2 (V617F) (%)	85 (76.6)	49 (76.6)	36 (76.6)	>0.95
Wild-Type JAK2 (%)	26 (23.4)	15 (23.4)	11 (23.4)	
Median Hb (g/dL) (IQR)	10.3 (8.9–12.0)	10.5 (9.3–12.4)	9.9 (8.8–11.2)	0.097
Median WBC (×10^9^/L) (IQR)	10.5 (5.7–15.8)	10.5 (6.1–17.1)	10.5 (5.1–13.9)	0.29
Median PMN (×10^9^/L) (IQR)	6.9 (3.7–11.4)	6.8 (3.9–13.6)	7.3 (3.3–10.4)	0.34
Median PLTs (×10^9^/L) (IQR)	250.0 (154.0–438.0)	250.5 (138.3–416.5)	250.0 (156.0–450.0)	0.60
Median Blast-Cells in PB (%) (IQR)	1.0 (0.0–2.0)	0.0 (0.0–2.0)	1.0 (0.0–2.0)	0.29
Median Spleen Length from LCM (cm) (IQR)	7.0 (6.0–10.0)	6.5 (5.0–10.0)	7.0 (6.0–10.0)	0.87
LDH < 2 ULN	28 (25.2)	16 (25.0)	12 (25.5)	>0.95
LDH ≥ 2 ULN	83 (74.8)	48 (75.0)	35 (74.5)	
IPSS Intermediate-I (%)	12 (10.8)	8 (12.4)	4 (8.5)	0.80
IPSS Intermediate-II (%)	35 (31.5)	20 (31.3)	15 (31.9)	
IPSS High Risk (%)	64 (57.7)	36 (56.3)	28 (59.6)	
DIPSS Intermediate-I (%)	36 (32.4)	22 (34.4)	14 (29.8)	0.66
DIPSS Intermediate-II (%)	49 (44.1)	29 (45.3)	20 (42.5)	
DIPSS High Risk (%)	26 (23.4)	13 (20.3)	13 (27.7)	
aaDIPSS Low Risk (%)	12 (10.8)	8 (12.5)	4 (8.5)	0.91
aaDIPSS Intermediate-I Risk (%)	35 (31.5)	20 (31.3)	15 (31.9)	
aaDIPSS Intermediate-II Risk (%)	31 (27.9)	18 (28.1)	13 (27.7)	
aaDIPSS High Risk (%)	33 (29.8)	18 (28.1)	15 (31.9)	
Median Time (months) from MF Diagnosis to Start of RUX (IQR)	7.6 (2.0–48.8)	5.0 (1.7–30.6)	14.4 (2.7–68.4)	0.071
RUX Initial Dosage:				0.87
5 mg BID (%)	16 (14.5)	8 (12.5)	8 (17.0)	
10 mg BID (%)	22 (19.8)	12 (18.8)	10 (21.3)	
15 mg BID (%)	27 (24.3)	16 (25.0)	11 (23.4)	
20 mg BID (%)	46 (41.4)	28 (43.7)	18 (38.3)	

Abbreviations: RUX, ruxolitinib; PMF, primary myelofibrosis; SMF, secondary myelofibrosis; PV-SMF, post-polycythemia vera MF; ET-SMF, post-essential thrombocythemia MF; Hb, hemoglobin; WBC, white blood cells; PMN, polymorphonuclear neutrophil; PLTs, platelets; PB, peripheral blood; LCM, left costal margin; LDH, lactate dehydrogenase; ULN, upper normal limit; IPSS, International Prognostic Score System; DIPSS, Dynamic International Prognostic Score System; aaDIPSS, age-adjusted Dynamic International Prognostic Score System; BID, twice daily; IQR, interquartile range.

## Data Availability

The data that support the findings of this study are available in the text and from the corresponding author, Massimo Breccia, upon reasonable request.

## Supplementary Materials

The following supporting information can be downloaded at https://www.mdpi.com/article/10.3390/jcm14196811/s1, Figure S1: Patient disposition flowchart; Table S1: Overall ruxolitinib (RUX) dosage at 3 and 6 months and differences in patients under or over 65 years old; Table S2: Ruxolitinib (RUX) dosage at 3 and 6 months in patients aged ≥65 years and differences in patients aged 65–74 years and over 75 years; Table S3: Distribution of hematological toxicity (drug-related anemia and drug-related thrombocytopenia) at 3 and 6 months from ruxolitinib (RUX) therapy initiation in patients aged 65–74 years and ≥75 years.

## Abbreviations

The following abbreviations are used in this manuscript:

MF	Myelofibrosis
PMF	Primary myelofibrosis
SMF	Secondary myelofibrosis
ET	Essential thrombocythemia
PV	Polycythemia vera
MPN	Myeloproliferative neoplasm
BM	Bone marrow
JAK2	Janus kinase 2
CALR	Calreticulin
MPL	Myeloproliferative leukemia virus oncogene/thrombopoietin receptor
OS	Overall survival
AML	Acute myeloid leukemia
IPSS	International Prognostic Scoring System
DIPSS	Dynamic International Prognostic Scoring System
aaDIPSS	Age-Adjusted Dynamic International Prognostic Scoring System
JAKi	Janus kinase inhibitors
RUX	Ruxolitinib
LCM	Left costal margin
PLTs	Platelet
Hb	Hemoglobin
RBC	Red blood cell
NCI-CTCAE	National Cancer Institute Common Terminology Criteria for Adverse Events
IWG-MRT	International Working Group on Myelofibrosis Research and Treatment
ELN	European LeukemiaNet
RR6	Response to Ruxolitinib After 6 Months
WHO	World Health Organization
IQR	Interquartile range
OR	Odds ratio
HR	Hazard ratio
CI	Confidence interval
AEs	Adverse events
