# Supplementary material for: Increased Rate of Anemia and Discontinuation in Older Patients with Myelofibrosis Treated with Ruxolitinib
Source: J Clin Med. 2025 Sep 26;14(19):6811. doi: 10.3390/jcm14196811 (PMC12524875; doi:10.3390/jcm14196811)
Supplement: Supplementary file 1 [file jcm-14-06811-s001.zip › jcm-3820304-supplementary.pdf]

Increased rate of anemia and discontinuation in older patients with myelofibrosis treated with Ruxolitinib

Alessandro Laganà[1], Emilia Scalzulli[1], Ida Carmosino[1], Maria Laura Bisegna[1], Claudia Ielo[1], Costanza Andriola[1], Maurizio Martelli[1], Massimo Breccia[1]

1. Hematology, Department of Translational and Precision Medicine, Policlinico Umberto I-Sapienza University, Rome, Italy

SUPPLEMENTARY FIGURES and TABLEs

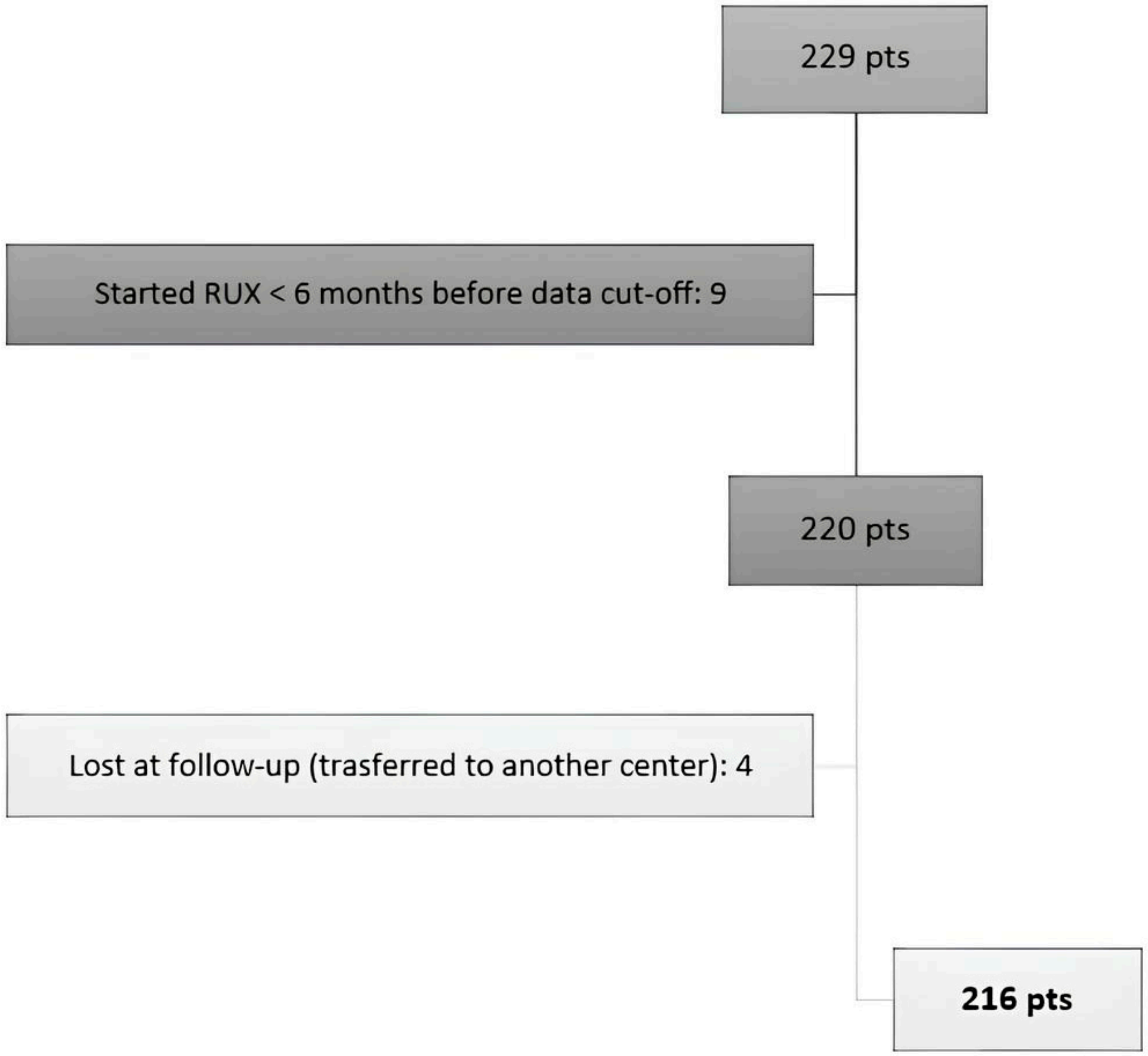

Supplementary Figure S1. Patient disposition flowchart. Diagram illustrating the total number of patients in the database and the number excluded from the analysis, along with the specific reasons for exclusion based on the study's selection criteria. Pts, patients; RUX, ruxolitinib.

Ruxolitinib Dosage	Total (N=216)	Age at RUX-Start < 65 years (N=105)	Age at RUX-Start ≥ 65 years (N=111)	p Value
RUX Dosage at 3 months:<br>5 mg daily (%)<br>5 mg BID (%)<br>15 mg daily<br>10 mg BID (%)<br>15 mg BID (%)<br>20 mg BID (%)	 4 (1.8)<br>39 (18.1)<br>18 (8.3)<br>54 (25.0)<br>49 (22.7)<br>52 (24.1)	 1 (1.0)<br>11 (10.5)<br>7 (6.7)<br>27 (25.7)<br>26 (24.7)<br>33 (31.4)	 3 (2.7)<br>28 (25.2)<br>11 (9.9)<br>27 (24.3)<br>23 (20.7)<br>19 (17.2)	**0.024**
RUX Dosage at 3 months:<br>≥ 20 mg daily (%)<br>< 20 mg daily (%)	 155 (71.8)<br>61 (28.2)	 86 (81.9)<br>19 (18.1)	 69 (62.2)<br>42 (37.8)	**0.002**
RUX Dosage at 3 months inferior to RUX Starting dosage (%)	104 (48.2)	52 (49.5)	52 (46.8)	0.79
RUX Dosage at 6 months:<br>5 mg daily (%)<br>5 mg BID (%)<br>15 mg daily<br>10 mg BID (%)<br>15 mg BID (%)<br>20 mg BID (%)	 7 (3.2)<br>47 (21.8)<br>16 (7.4)<br>58 (26.8)<br>41 (19.0)<br>47 (21.8)	 2 (1.9)<br>13 (12.4)<br>6 (5.7)<br>32 (30.5)<br>21 (20.0)<br>31 (29.5)	 5 (4.5)<br>34 (30.6)<br>10 (9.1)<br>26 (23.4)<br>20 (18.0)<br>16 (14.4)	**0.007**
RUX Dosage at 6 months:<br>≥ 20 mg daily (%)<br>< 20 mg daily (%)	 146 (67.6)<br>70 (32.4)	 84 (80.0)<br>21 (20.0)	 62 (55.9)<br>49 (44.1)	**< 0.001**
RUX Dosage at 6 months inferior to RUX Starting dosage (%)	112 (51.6)	55 (51.9)	57 (51.4)	0.89
RUX Dosage at 6 months inferior to RUX dosage at 3 months (%)	36 (16.7)	13 (12.5)	23 (20.7)	0.14

Supplementary Table S1. Overall ruxolitinib (RUX) dosage at 3 and 6 months and differences in patients under or over 65-year old. RUX, ruxolitinib; BID, twice daily.

Ruxolitinib Dosage	Age at RUX-Start ≥ 65 years (N=111)	Age at RUX-Start 65-74 years (N=64)	Age at RUX-Start ≥ 75 years (N=47)	p Value
RUX Dosage at 3 months:<br>5 mg daily (%)<br>5 mg BID (%)<br>15 mg daily<br>10 mg BID (%)<br>15 mg BID (%)<br>20 mg BID (%)	 3 (2.7)<br>28 (25.2)<br>11 (9.9)<br>27 (24.3)<br>23 (20.7)<br>19 (17.2)	 2 (3.1)<br>17 (26.6)<br>8 (12.5)<br>13 (20.3)<br>16 (25.0)<br>8 (12.5)	 1 (2.1)<br>11 (23.4)<br>3 (6.4)<br>14 (29.8)<br>7 (14.9)<br>11 (23.4)	0.36
RUX Dosage at 3 months:<br>≥ 20 mg daily (%)<br>< 20 mg daily (%)	 69 (62.2)<br>42 (37.8)	 37 (57.8)<br>27 (42.2)	 32 (68.1)<br>15 (31.9)	0.32
RUX Dosage at 3 months inferior to RUX Starting dosage (%)	52 (46.8)	33 (51.6)	19 (40.4)	0.26
RUX Dosage at 6 months:<br>5 mg daily (%)<br>5 mg BID (%)<br>15 mg daily<br>10 mg BID (%)<br>15 mg BID (%)<br>20 mg BID (%)	 5 (4.5)<br>34 (30.6)<br>10 (9.1)<br>26 (23.4)<br>20 (18.0)<br>16 (14.4)	 3 (4.7)<br>15 (23.4)<br>5 (7.8)<br>18 (28.2)<br>16 (25.0)<br>7 (10.9)	 2 (4.3)<br>19 (40.4)<br>5 (10.6)<br>8 (17.1)<br>4 (8.5)<br>9 (19.1)	0.13
RUX Dosage at 6 months:<br>≥ 20 mg daily (%)<br>< 20 mg daily (%)	 62 (55.9)<br>49 (44.1)	 41 (64.1)<br>23 (35.9)	 21 (44.7)<br>26 (55.3)	0.060
RUX Dosage at 6 months inferior to RUX Starting dosage (%)	57 (51.4)	33 (51.6)	24 (51.1)	> 0.95
RUX Dosage at 6 months inferior to RUX dosage at 3 months (%)	23 (20.7)	7 (10.9)	16 (34.0)	**0.004**

Supplementary Table S2. Ruxolitinib (RUX) dosage at 3 and 6 months in patients aged ≥ 65 years and differences in patients aged 65-74 years and over 75-year old. RUX, ruxolitinib; BID, twice daily.

	Drug-related Anemia			Drug-related Thrombocytopenia		
	Age at RUX-Start 65-74 years (N=64)	Age at RUX-Start ≥ 75 years (N=47)	p Value	Age at RUX-Start 65-74 years (N=64)	Age at RUX-Start ≥ 75 years (N=47)	p Value
**At 3 months (%)**	27 (42.2)	24 (51.1)	0.44	9 (14.1)	6 (12.8)	> 0.95
New RBC transfusion dependency at 3 months (%)	14/50 (28.0)	12/34 (35.3)	0.48	-	-	-
**At 6 months (%)**	25 (39.1)	20 (42.6)	0.85	10 (15.6)	7 (14.9)	> 0.95
New RBC transfusion dependency at 6 months (%)	10/50 (20.0)	14/34 (41.2)	**0.049**	-	-	-

Supplementary Table S3. Distribution of haematological toxicity (drug-related anemia and drug-related thrombocytopenia) at 3 and 6 months from ruxolitinib (RUX) therapy initiation in patients aged 65-74 years and ≥ 75 years.
